# Special Issue on “Ubiquitin and Autophagy”

**DOI:** 10.3390/cells10010116

**Published:** 2021-01-10

**Authors:** Taras Y. Nazarko

**Affiliations:** Department of Biology, Georgia State University, Atlanta, GA 30303, USA; tnazarko@gsu.edu; Tel.: +1-404-413-5349

The Special Issue of *Cells* on “Ubiquitin and Autophagy” is a tribute to the multifaceted role of ubiquitin and autophagic ubiquitin-like (UBL) proteins in the autophagy-related (ATG) pathways. Ubiquitin is a small regulatory protein that is used to modify other proteins in the process called ubiquitination. The specificity of ubiquitination depends on ubiquitin ligases, the enzymes that place ubiquitin on specific substrates. They are counteracted by ubiquitin proteases that perform deubiquitination. As a result of ubiquitination of a substrate and ubiquitination of ubiquitin itself, proteins become polyubiquitinated with various ubiquitin chains and degraded via the ubiquitin-proteasome system (UPS), autophagy-lysosomal pathway or endo-lysosomal pathway. The polyubiquitination of proteins in protein aggregates and at the surface of organelles or intracellular pathogens often tags these subcellular structures for sequestration (by the double-membrane vesicles, autophagosomes) and delivery to the lysosomes for degradation and recycling by the diverse selective autophagy pathways.

The “Ubiquitin and Autophagy” Special Issue features 13 papers: seven research articles [1,2,3,4,5,6,7] and six reviews [8,9,10,11,12,13]. All of them are at the intersection of ubiquitin-related processes and autophagy, including the roles of: (1) ATG and UBL proteins in the UPS and autophagy [2,3], (2) ubiquitin-binding autophagic receptor, p62, in autophagy signaling [4], (3) LC3-interacting region (LIR) of connexins in binding to UBL proteins [6], (4) proteasomal deubiquitinating enzyme, PSMD14, in autophagy [7], (5) sorting nexins in the UPS, autophagy and endocytosis [8], (6) ubiquitin and UBL proteins in selective autophagy [9], as well as (7) structures and interactions of the UBL proteins of Atg8-family [13]. In addition to the studies on ubiquitin and autophagy in cell culture, several groups used model organisms, such as laboratory mice [1,5], nematode *Caenorhabditis elegans* [2], and social amoeba *Dictyostelium discoideum* [3]. Besides, the review article of Ma and colleagues discusses the interplay between the UPS and autophagy in plants [10]. Although most of the studies explored the fundamental molecular and cellular mechanisms, some of them have interesting implications for human diseases, such as cancer [4] and leukodystrophy [5]. On top of that, the review article of Watanabe et al. is dedicated to the role of ubiquitin and autophagy in neurodegenerative diseases [12].

Given a complex relationship between the UPS and autophagy that started to be appreciated recently (and was one of the motivations for this Special Issue), it is not surprising that many research articles have a common sub-theme of “UPS-autophagy crosstalk”. For example, Kang et al. reported that decreased proteasomal degradation of TonEBP protein under the ER stress conditions in β-cells is responsible for increased autophagosome formation, decreased accumulation of protein aggregates and better cell survival [1]. However, the decreased cleavage of K63-ubiquitin chains by proteasomal PSMD14 promotes retention of ATG9A and RAB1A proteins in Golgi apparatus and blocks autophagy via the reduced Golgi-to-ER retrograde transport [7]. Furthermore, deficient autophagy due to knockdown or knockout of the genes encoding ATG and UBL proteins in *C. elegans* and *D. discoideum* negatively affects the UPS suggesting that UPS is not always compensating for the lack of autophagy in vivo. Moreover, a fully functional UPS might depend on autophagy in the tissue- and organism-specific manner [2,3]. Interestingly, Lin et al. described the impairment of both the UPS and autophagy in the cellular and murine (twitcher mice) models of globoid cell leukodystrophy [5].

Another underlying sub-theme of the issue revolves around p62 and its functions. While p62 serves as a ubiquitin-binding receptor for many selective autophagy pathways and accumulates on the ubiquitin-positive aggregates in the brains of twitcher mice [5], it also negatively regulates TLR4 signaling and autophagy by disrupting the intermediate TRAF6-BECN1 signaling complex and inhibiting BECN1 ubiquitination in cancer cells what reduces their migration and invasion after TLR4 stimulation [4]. Finally, Catarino et al. reported an alternative, ubiquitin- and p62-independent, mechanism for bridging connexins with the UBL proteins of Atg8-family, LC3B and GABARAP. The direct binding of connexin, Cx43, to LC3/GABARAP proteins via its LIR motif ensures efficient degradation of Cx43 by the p62-mediated selective autophagy [6]. 

Many researchers from the ubiquitin and autophagy fields will find this issue interesting either due to the thought-provoking original findings or thoughtful summaries of the literature. If you are new to the field or look for a general overview on the theme of “Ubiquitin and Autophagy” before diving into the more specific area of research, the review article of Klionsky and colleagues discusses a wide range of topics, including the autophagic UBL conjugation systems, UPS-autophagy interplay, ubiquitin signaling in selective autophagy, and autophagy regulation by ubiquitination/deubiquitination of the main players [11]. Therefore, this can serve as a starting point. I hope you enjoy reading our collection of papers on “Ubiquitin and Autophagy”, and share my excitement about new developments at the intersection of ubiquitin and autophagy fields.
